# Management or technology? Firms’ embeddedness in dual talent flow networks and their innovation performance

**DOI:** 10.3389/fpsyg.2022.1028818

**Published:** 2022-11-08

**Authors:** Bo Sun, Ao Ruan, Biyu Peng, Shanshi Liu

**Affiliations:** ^1^School of Economics and Management, South China Normal University, Guangzhou, China; ^2^International Business School, South China Normal University, Guangzhou, China; ^3^School of Business Administration, South China University of Technology, Guangzhou, China

**Keywords:** dual networks, management talent flow, technical talent flow, firms’ innovation performance, resume data

## Abstract

This paper examines the complex relationship between different types of talent flow networks and firms’ innovation. Based on the social network theory and human capital theory, we divide the talent flow networks into “management talent flow networks” and “technical talent flow networks”. The paper then investigates the potential interacting effect and matching effect between the two types of networks when they influence the innovation of firms. The empirical results, which draw from LinkedIn (China) resume data show that: (1) in both management talent flow networks and technical talent flow networks, higher degree of centrality and larger structural hole indexes can enhance firms’ innovation performance; (2) there is significant interacting effect between management talent flow networks and technical talent flow networks in their influence on firms’ innovation. That is, the interaction between firms’ centrality in management talent flow networks and technical talent flow networks, and the interaction between firms’ structural hole indexes in the two networks can both enhance their innovation performance; (3) there is also noteworthy matching effect between the two network types. That is, firms with balanced degree centrality (high-high, or low-low) and balanced structural hole indexes (high-high, or low-low) in management talent flow networks and technical talent flow networks exhibit better innovation performance than those with imbalanced degree centrality (high-low, or low-high) and structural hole indexes (high-low, or low-high) in the two networks. This paper contributes to the classification research on talent flow networks, and deepens our understanding of the complex influencing mechanism between talent flow networks and firms’ innovation. Moreover, it provides managerial implications for firms to improve innovation performance *via* talent flow management.

## Introduction

Innovation is acknowledged as a crucial business activity for firms to gain core competitive power (Crossan and Apaydin, 2009; Rajapathirana and Hui, 2018). However, as the innovation process grows more complicated, it becomes challenging for individual firms to maintain continuous innovation with their limited resources including information, knowledge and technology (Dahlander and Gann, 2010). Hence, firms need to break the organizational boundaries for external innovative resources (Stefan and Bengtsson, 2017). In recent years, an increasing number of studies have asserted that the inter-organizational networks are important channels for firms to acquire key innovative resources from the outside (Pittaway et al., 2004; Provan et al., 2007). In this context, the researchers have proposed many interorganizational networks such as alliance network (Gulati, 1998), collaboration network (Ahuja, 2000) and talent flow network (Dokko and Rosenkopf, 2010; Shipilov et al., 2017).

Talent flow networks are particularly noteworthy these are social relation networks shaped by the mobility of talent between organizations (Godart et al., 2014; Shipilov et al., 2017). Some of the existing literature states that, by occupying advantageous positions in the talent flow network, firms can obtain abundant high-quality innovative resources consisting of heterogeneous information, knowledge and technology, which may help to foster better innovative performance (Shipilov et al., 2017; Sun et al., 2022). Comparatively, when firms base their resource acquisition strategies on talent flow, they may gain innovative resources including core knowledge and information from the external organizations more directly and deeply (Song and Wu, 2003; Godart et al., 2014; Shipilov et al., 2017; Sun et al., 2022).

The influence of talent flow network on firms’ innovation performance has been gaining increasing interest in the research community. However, most of the existing literature places emphasis on how the technical talent flow networks affects firms’ innovation (Dokko and Rosenkopf, 2010; Godart et al., 2014; Shipilov et al., 2017) while overlooking the effect of management talent flow networks and other non-technical networks. The complex relationship between these networks in the influencing process has also been neglected. As such, our understanding about the influencing mechanism between talent flow network and firms’ innovation may be incomplete. As suggested by the total innovation model, innovation is generated by the combined effect of technical and non-technical factors (Xu et al., 2007). Although the technical factor plays the pivotal role, the non-technical factors have important effect as well. There may also be synergic or substitutional relationship between them (Daft, 1978; Tang, 1998; Tidd et al., 2005; Xu et al., 2007). For example, management talent may bring non-technical innovative resources, such as managerial knowledge and market information, which can help firms making decisions and allocating resources more reasonably and efficiently, thereby enhancing firms’ success rate when transforming the technical innovative resources into innovation performance. To address the current research gaps, this paper investigates the effects of both technical talent and management talent on firms’ innovation. In view of this, the research questions are as follow: (1) how do technical talent flow networks and management talent flow networks influence the innovation of firms? (2) How are the two types of networks related to each other in their influence over firms’ innovation?

This paper attempts to investigate the impact of talent flow networks on firms’ innovation more deeply based on the existing researches. From the perspective of network content, we divide the talent flow networks into management talent flow networks and technical talent flow networks according to the profession category of talent. Then, we try to analyze the diverse and complex effect of the two types of networks on firms’ innovation. To obtain valid data-set for our research, we first collected the online resume data from LinkedIn (China) per the method outlined by Ge et al. (2016). Next, with reference to Dokko and Rosenkopf (2010), we set up and measured the inter-organizational networks for management talent and technical talent shaped by inflow of them. Finally, using firm names, we matched the listed companies to the business data from China Stock Market & Accounting Research Database (CSMAR), and obtained a panel data-set with a time interval from 2000 to 2015.

The empirical results show that: (1) both management talent flow networks and technical talent flow networks have significant positive effects on firms’ innovation. That is, when firms possess higher degree of centrality and more structural holes in the two networks, their innovation performance improves; (2) there is significant interacting effect between management talent flow networks and technical talent flow networks when they influence firms’ innovation, which means that, the interaction between firms’ degree centrality in management talent flow networks and technical talent flow networks and the interaction between firms’ structural hole indexes in the two networks can both enhance their innovation performance; (3) In terms of network embedding, firms that can balance their degree centrality and structural holes in the the two networks will achieve better innovation performance compared to those that can not maintain the balance. That is, firms that embed evenly into the two networks may achieve better innovation performance compared to those who embed unevenly. More specifically, firms with balanced degree centrality (high-high, or low-low) in the two networks may attain superior innovation performance compared to those with imbalanced degree centrality (high-low, or low-high). Similarly, firms with balanced structural hole indexes (high-high, or low-low) in the two networks may achieve better innovation than those with imbalanced structural hole indexes (high-low, or low-high).

The rest of the paper is organized as follow: Section 2 presents relevant theories and develops the main research hypotheses; Section 3 introduces the research design including the sources of data, the selection of samples, the explanation of variables and the development of regression models; Section 4 presents our analysis of the regression results; Section 5 concludes the paper with a discussion; Section 6 presents the study’s limitations and suggestions for future research.

## Theories and hypotheses

### Firms’ embeddedness in dual talent flow networks

Talent flow networks that are shaped by the mobility of different types of talent usually carry different resources. Hence, the potential impact of these networks on firms’ strategic behaviors and performance also varies (Hillman and Dalziel, 2003; Shipilov et al., 2017).

Management talent and technical talent are the two main categories of talent. Thus, we can divide the talent flow networks into “management talent flow networks” and “technical talent flow networks.” Dual embeddedness means that firms get embedded simultaneously in the two types of networks. The network for management talent is an inter-organizational social network shaped by the flow of management talent. It mainly carries the migration of management human capital resources, including non-technical innovative resources such as management knowledge, experience and customer market information. For this type of network, the key to promoting firms’ innovation is to improve their decision-making efficiency for innovation and enhance the internal management level (Talke et al., 2010; Clark and Maggitti, 2012). As for technical talent flow networks, it is an inter-organizational social network formed by the inflow of technical talent. It mainly reflects the mobility of technical human capital resources, consisting of technical innovative resources such as product design, product development, and the cutting-edge technological information. This type of network enhances firms’ innovation mainly by raising the capability of product research and development (R&D; Shipilov et al., 2017).

Firms’ innovation performance is the result of the combined effects of the two types of talent flow networks. According to the total innovation management model, the realization of innovation is supported not only by the technical innovative system, but also by the interworking between technical factors and the other non-technical factors such as strategy, management and marketing (Tang, 1998; Tidd et al., 2005; Xu et al., 2007). The matching and collaboration between management and technical factors are particularly important (Daft, 1978). When firms try to achieve innovation through talent, they need to balance their embeddedness in the network for management talent and that for technical ones, thereby balancing the acquisition of management and technical human capital resources.

### Dual talent flow networks and firms’ innovation

Firms that occupy advantageous positions in the management talent flow networks can acquire abundant, non-redundant and high-quality management experience and knowledge, as well as market information and management talent. Specifically, firms taking the central positions in the network can establish adequate connections with external organizations, through which they may obtain substantial management knowledge and experience, as well as consumer market information and management talent (Casper, 2007; Rogan and Mors, 2017; Broschak et al., 2020). Similarly, firms possessing more structural holes in the network may obtain more high-quality, non-redundant connections with the other organizations. This help the firms obtain information and knowledge resources from the network more quickly. More importantly, the resources they obtain are characterized by a higher degree of heterogeneity (Burt, 1992; Aral and Van Alstyne, 2011; Phelps et al., 2012).

This paper asserts that, those abundant, non-redundant high quality management resources can influence firms’ innovation in both direct and indirect ways. First, high quality market information and management talent help firms identify the R&D areas with good market prospects (Crossan and Apaydin, 2009; Zhou and Li, 2012). This helps the firms make quick and clear strategic investment decisions for innovation (Clark and Maggitti, 2012), which directly enhances their innovation. Moreover, management talent flow networks bring management talent, knowledge and experience that may improve firms’ innovation indirectly by influencing their innovation culture and resource allocations. Specifically, firms that encourage innovation and learning (Damanpour, 1991; West and Anderson, 1992) tend to attract technical talent and R&D talent who will design and develop innovative products more adventurously. This promotes communication and learning within firms, which may lead to more innovative ideas and solutions. Meanwhile, the high-level management talent can plan the existing financial resources (Parthasarthy and Hammond, 2002) and human resources (Crossan and Apaydin, 2009) more reasonably. They can also optimize the utilization of redundant resources (Damanpour, 1991). Finally, the R&D projects will be supported more efficiently within firms.

The above discussion leads to the following hypotheses:

*Hypothesis 1*: Occupying advantage positions in the management talent flow networks has positive effect on firms’ innovation.

*Hypothesis 1a*: When firms have higher degree of centrality in the management talent flow networks, their innovation performance will improve.

*Hypothesis 1b*: When firms possess more structural holes in the management talent flow networks, their innovation performance will improve.

For firms, developing innovative products is a complicated process. It comprises various activities, such as idea generation, idea elaboration, idea championing, and idea implementation (Perrysmith and Mannucci, 2017). The focus of these innovative activities and the resources they require differ with each development stage. Idea generation and elaboration belong to the orienting stage of the process, when firms need more information about the consumer market demand and the frontier R&D technologies so that they can quickly determine practicable and marketable R&D directions. Idea championing and implementation are part of the practice stage, when firms need more professional R&D technology and knowledge to overcome technical difficulties.

This paper suggests that, firms that occupy superior positions in the technical talent flow networks have advantages when acquiring and controlling the heterogeneous information, knowledge and technology resources. Such positions benefit firms’ innovation performance by speeding up the determination of innovation directions and solving R&D obstacles. Furthermore, Firms occupying central positions in the technical talent flow networks have more sufficient connections with the other organizations. This means that they can obtain more abundant information about new product development and frontier technologies through the mobility of technical talent (Dokko and Rosenkopf, 2010; Godart et al., 2014; Shipilov et al., 2017). Meanwhile, firms that possess more structural holes in the technical talent flow networks can establish more high quality and non-redundant connections with the other firms, thereby obtaining diverse frontier knowledge, information and technologies (Burt, 1992; Aral and Van Alstyne, 2011; Phelps et al., 2012).

These cutting-edge innovative resources will play different roles at different stages of the innovative process, ultimately promoting the overall innovation performance of firms. Specifically, on the one hand, the non-redundant technological information from different industries and product demand information from various markets (Dokko and Rosenkopf, 2010; Wang et al., 2017) facilitate the rapid recognition of marketable technology frontier and trend (Lingo and Omahony, 2010). This helps firms identify the marketable product R&D directions faster (Wang et al., 2017), thus expediting idea generation and elaboration. Similarly, the acquisition of plentiful non-redundant R&D technologies help firms overcome technical problems and R&D bottlenecks (Perrysmith and Mannucci, 2017; Wal et al., 2020), thereby speeding up the idea championing and idea implementation. Some of the existing empirical research verifies that holding advantageous positions in the technical talent flow networks has positive effect on firms’ innovation performance (Godart et al., 2014; Shipilov et al., 2017).

Based on the above observations, this paper proposes the following hypotheses:

*Hypothesis 2*: Holding superior positions in the technical talent flow networks has positive impact on firms’ innovation performance.

*Hypothesis 2a*: When firms have higher degree of centrality in the technical talent flow networks, their innovation performance will improve.

*Hypothesis 2b*: When firms have more structural holes in the technical talent flow networks, their innovation performance will improve.

### Interaction of dual talent flow networks and firms’ innovation

The technical talent flow networks influences firms’ innovation mainly by promoting their R&D capabilities. However, during the product development process, the R&D group may encounter problems such as lack of innovative resources or constraints from the internal management system (Damanpour, 2014). Such problems may weaken the positive effect of the technical talent flow networks. When firms occupy advantage positions, such as structural holes and nodes near the center of the management talent flow networks, they can access to heterogeneous and deep-level resources including market information, business knowledge and management experiences from the external organizations (Casper, 2007; Rogan and Mors, 2017; Broschak et al., 2020). These resources may contribute to the improvement of firms’ strategic decision-making, internal relationship collaboration and resource management, as well as help the R&D teams solve the problems of resource shortage and management constraints, thereby enhancing the positive effect of the technical talent flow networks on firms’ innovation.

Practically speaking, the acquisition of adequate market information can help firms avoid potential risks and identify those commercially promising R&D directions (Roberts and Grover, 2012; Roberts et al., 2016). The firms can then reasonably allocate more human resources and financial resources to these directions (Hollen et al., 2013). This may facilitate the success and commercialization of their R&D activities. Moreover, rich business knowledge and management experiences will improve firms’ capability in strategic adjustment and internal relation collaborations (Cohen and Levinthal, 1990), which helps to overcome the constraints caused by organizational structures and administrative regulations (Hollen et al., 2013). This helps guarantee the high efficiency of the R&D activities.

The management talent flow networks affect firms’ innovation mostly by influencing their R&D investment decisions. When firms allocate resources to different R&D projects, the top management team’s investment decisions might be inefficient and ineffective because it could be difficult for them to discern the trends and prospect of technology development (Roberts et al., 2016). This can weaken the positive effect of the management talent flow network. If firms possess advantageous nodes and structures in the technical talent flow networks, they will be able to acquire deep-tier and diversified technical information, product knowledge and R&D technologies through the inflow of technical talent (Dokko and Rosenkopf, 2010; Godart et al., 2014; Shipilov et al., 2017). And with the external information about technological development, firms can increase their accumulation of product development knowledge and technologies, which helps the management team recognize the promising technological fields and promote the efficiency and effectiveness of their R&D input decisions (Clark and Maggitti, 2012). This can strengthen the positive effect of the management talent flow on innovation.

The above observations lead to the following hypotheses:

*Hypothesis 3*: Management talent flow networks and technical talent flow networks interactively enhance firms’ innovation.

*Hypothesis 3a*: Firms’ degree of centrality in management talent flow networks and technical talent flow networks enhance their innovation performance interactively.

*Hypothesis 3b*: Firms’ structural hole indexes in management talent flow networks and technical talent flow networks are interactively associated with better innovation.

### The matching effect of dual talent flow networks and firms’ innovation

According to the law of diminishing marginal utility, with the other terms remain unchanged, when the allocation of one productive factor is too high, the marginal revenue of it will become less than its marginal cost, thus bringing down the marginal benefit. The existing innovation theories state that, firms’ innovations rely on the collaboration of different technological, market and management factors (Tidd et al., 2005). Hence, this paper infers that, firms need to balance the acquisition of technical and management resources while enhancing their innovation by obtaining innovative resources from the management talent flow networks and the technical talent flow networks. They should embed into the two networks equally according to a ratio considering the embedding structures and positions in the networks.

Specifically, if firms over-stress their embeddedness in the management talent flow networks while overlooking the technical talent flow networks, they may be able to improve their management and resource allocation efficiency with the management human capital resources to some extent. However, due to the shortage of technical human capital resources, they can hardly solve the technical problems that their R&D team will encounter during the product development process. Similarly, when firms overemphasize the embeddedness in the technical talent flow networks while underestimating the management ones, they may be able to propose better ideas, and provide proper solutions to specific technical problems. But for the R&D staff, the lack of management human capital resources may lead to inadequate resource allocation and insufficient administrative support.

These observations indicate that firms must embed into the two networks evenly. This allows them to maintain the R&D capacity with the resources from the technical talent flow networks, while maintaining the decision-making efficiency with the resources from the management talent flow networks. Then firms’ innovation performance can be promoted efficiently and effectively.

The above discussions contribute to the following hypotheses:

*Hypothesis 4*: Balanced embeddedness in the management talent flow networks and technical talent flow networks has positive impact on firms’ innovation.

*Hypothesis 4a*: Balanced degree centrality in the management talent flow networks and technical talent flow networks is positively associated with firms’ innovation.

*Hypothesis 4b*: Balanced structural holes in the management talent flow networks and technical talent flow networks is positively associated with firms’ innovation.

## Research design

### Sample selection and data sources

This study selects data samples from the public resume data on LinkedIn (China) and the listed company data from CSMAR for the time period between 2000 and 2015. It is noteworthy that, Resume data from 2016 onward were unavailable at the time of collection due to policies in China. While this means we were unable to update the data to the most recent year. This keeps the current study in line with related studies, both domestic and foreign, which typically base their researches on historical data (Dokko and Rosenkopf, 2010; Ge et al., 2016; Shipilov et al., 2017).

The detailed data collection process is as follows: first, we used the method outlined by Ge et al. (2016) to acquire job mobility data from LinkedIn (China) with web crawlers. Then, referring to Dokko and Rosenkopf (2010), we used Pajek 3.0 to build up the management talent flow networks and the technical talent flow networks which are shaped by inter-organizational mobility of management talent and technical talent. We then calculate the two measuring indexes, degree centrality and structural hole index, to represent the advantageous positions and structures in the two networks. Next, we integrated the network structure data-set and the CSMAR financial data-set according to year and the listed company code. Finally, an unbalanced panel data-set for the time period from 2000 to 2015 is obtained. The data-set contains firm name, degree centrality, structural hole index, firms’ inventions and patents, and a number of control variables.

### The main variables

#### Advantageous positions and structures in the talent flow networks

##### Degree centrality

To address the discrepancy of network size in different years, we standardize the degree centrality with the method outlined by Dokko and Rosenkopf (2010). Then we set up the calculation for firms’ degree centrality in the talent flow networks. As can be seen in equation (1), *i* represents a certain firm of a certain year in the sample, while *j* represents any other firms except firm i in the same year. *X_ji_ represent*s the talent flow from firm *j* to firm *i* (we only count the occurrence of the flow, not the quantity. So the value is either “0” or “1”). Finally, *g* represents the number of firms in the sample of the same year.


(1) $$DC_{i}=\frac{\underset{j}{\sum }X_{ji}}{\left(g-1\right)}$$

##### The structural hole indexes

Per the process outlined by Burt (1992), we set up the calculation formulas for the structural hole indexes. As illustrated in equations (2), (3) and (4), *i* represents a certain firm of a certain year in the sample, while *j* represents any other firms except i. *SHD_i_* is the structural hole index of firm *i*. *C_i_* is the restraint coefficient of firm *i* in the network, indicating the degree of firm *i’s* direct connection and indirect connection with the other firms in the sample. Larger *C_i_* means that firm i has stronger connections with the other firms, and fewer structural holes in the talent flow network. As the maximum value of *C_i_* is 1, we use 1 minus *C_i_* to measure the structural hole index of firm *i* in the network. Besides, *P_ij_* represents the direct connections shaped by talent flow between firm *i* and firm *j*, while $\underset{q}{\sum }P_{iq}P_{jq}$ represents the indirect connections between them.


(2) $$SHD_{i}=1-C_{i}$$

(3) $$C_{i}=\overset{n}{\underset{j=1}{\sum }}C_{ij},i\neq j$$

(4) $$C_{ij}={\left(p_{ij}+\underset{q}{\sum }p_{iq}p_{qj}\right)}^{2};q\neq i,j$$

#### Interactions and matching between talent flow networks

With the inflow of management and technical talent, firms can embed into the management talent flow networks and technical talent flow networks simultaneously (Dokko and Rosenkopf, 2010), thus presenting the network feature of dual embeddedness. According to the method outlined by Tsui et al. (1997), He and Wong (2004), we measure the interaction of the two networks *via* the product term of their structure indexes. Using the median of network structure index, we divide the two networks into the balanced embedding group (with high embedding indexes in both management and technical talent flow networks, or, with low embedding indexes in both networks) and an imbalanced embedding group (with a high embedding index in the management networks and a low embedding index in the technical one, or, with a low embedding index in the management one and a high embedding index in the technical one). This grouping represents the degree of embedding balance in the dual talent flow networks. Dual network interactions consist of the interaction of firms’ centrality (*MTD*) and structural holes (*MTS*) in the management and technical talent flow networks, while a balance of dual network embedding refers to the balance of firms’ centrality (*MTDB*) and structural holes (*MTSB*) in the two types of networks.

#### Firms’ innovation performance

Firms’ innovation is usually interpreted as the presentation of new products or new ideas. Using the method proposed by Ahuja (2000), this paper uses the logarithm of the sum of firms’ inventions and patents to represent their innovation performance.

#### Control variables

This study controls several variables that may exert an influence over firms’ innovation outcomes, such as firms’ profitability, growth capacity, size, investment opportunities and risk appetite. Accordingly, per Ahuja (2000)‘s method, we use the return of assets (*ROA*), the increase rate of business revenue (growth), the natural logarithm of the number of employees at year end (*Size*), the Tobin’s Q value (*Tobin’s Q*) and the debt to asset ratio (*Lev*) to control the above mentioned variables. Moreover, we set the year and industry dummies to control the effects of year and industry. The definitions of all variables are included in Table 1.

**Table 1:** Definitions of the main variables.

Variables	Symbol	Definitions
Innovation performance	*INNO_it_*	*INNO* represents the innovation performance of listed firms. Specifically, it is the natural logarithm of the sum of firms’ patents and inventions in year *t.*
Firms’ degree centrality in the talent flow network	*MDC_it_, TDC_it_*	Firms’ degree centrality in year *t*. *MDC* is the degree centrality in management talent flow network. *TDC* is the degree centrality for the technical one.
Firms’ structural hole index in the talent flow network	*MSHD_it_, TSHD_it_*	Firms’ structural hole index in year *t*. *MSHD* is the index in the management network. *TSHD* is the index in the technical network.
Dual network interactions	*MTD_it_，MTS_it_*	The interaction of firms’ degree centrality and structural holes in year *t. MTD* and *MTS* represent the interaction of degree centrality and structural holes in the management network and technical network, respectively.
Dual network matching	*MTDB_it_，MTSB_it_*	The balance of firms’ degree centrality and structural holes in year *t*. *MTDB* and *MTSB* represent the balance of degree centrality and structural holes in the management network and technical network, respectively.
Profitability	*ROA_it_*	Firms’ year-end total asset in year *t*.
Growth capacity	*Growth_it_*	Year-end total revenue in year *t-Year*-end total revenue in year *t-1*/ Year-end total revenue in year *t−1*
firm size	*Size_it_*	The natural logarithm of the year-end total number of employees in year *t.*
Firms’ future investment opportunities	*Tobin’s Q_it_*	Firms year-end market value in year *t* / Replacement cost of capital
The debt to asset ratio	*Lev_it_*	Total year-end liabilities/Total assets
Enterprise property	*Property_it_*	Dummies of enterprise property. 1 means state-owned enterprise. 0 means non-state-owned enterprise.
Industry attribute	*Industry_it_*	Industry dummy
Year	*Year_it_*	Year dummy

## Empirical results and analysis

### The descriptive statistics and correlation analyses

Table 2 lists the descriptive statistics and the correlation analyses of the main variables.

**Table 2:** The descriptive statistics and correlation analysis of the main variables.

	*Obs.*	*INNO*	*MDC*	*MSHD*	*TDC*	*TSHD*	*ROA*	*Growth*	*Size*	*Tobin’ Q*	*Lev*
*INNO*	1,625	1									
*MDC*	1,625	0.248^***^	1								
*MSHD*	1,625	0.297^***^	0.368^***^	1							
*TDC*	1,625	0.252^***^	0.947^***^	0.273^***^	1						
*TSHD*	1,625	0.320^***^	0.368^***^	0.705^***^	0.312^***^	1					
*ROA*	1,625	0.039^**^	−0.006	0.013	0.008	0.025	1				
*Growth*	1,625	−0.021	0.045^***^	0.047^***^	0.046^***^	0.039^**^	0.100^***^	1			
*Size*	1,625	0.413^***^	0.247^***^	0.384^***^	0.190^***^	0.356^***^	0.035^***^	0.043^***^	1		
*Tobin’ Q*	1,625	−0.143^***^	−0.090^***^	−0.039^***^	−0.062^***^	−0.009	0.044^***^	0.046^***^	−0.348^***^	1	
*Lev*	1,625	0.165^***^	0.186^***^	0.211^***^	0.123^***^	0.171^***^	−0.172^***^	0.055^***^	0.505^***^	−0.322^***^	1
*Mean*	—	3.168	0.001	0.440	0.001	0.416	0.049	0.194	22.300	2.014	0.466
Std.	—	1.594	0.003	0.377	0.004	0.382	0.065	0.319	1.859	1.271	0.207
Min	—	0.693	0	0	0	0	−0.728	−0.552	18.430	0.910	0.051
Max	—	8.752	0.039	0.996	0.070	1	0.482	2.573	28.690	8.270	0.977

Pearson correlation coefficients are in the table.

*** *p* < 0.01;

** *p* < 0.05;

* *p* < 0.1.

### Regression results

#### The influence of dual talent flow networks and the interactions between them over the innovation performance of firms

Table 3 reports the results of the main regression which examines the influence of management talent flow networks, technical talent flow networks, and their interactions on firms’ innovation performance. The regression tests how the advantageous positions and structures (the degree centrality and the structural hole indexes) in the two networks, and the interaction between the two networks, can interactively affect the innovation of firms. The regression results validate Hypotheses 1a, 1b, 2a, 2b, 3a and 3b.

**Table 3:** The interactive impact of dual talent flow network on firms’ innovation.

Variables	*INNO*				
	Column (1)	Column (2)	Column (3)	Column (4)	Column (5)
*MDC*		0.802^***^		−1.692^**^	
		(0.162)		(0.805)	
*MSHD*		0.381^***^			0.158
		(0.101)			(0.115)
*TDC*			0.599^***^	2.344^***^	
			(0.069)	(0.685)	
*TSHD*			0.464^***^		0.257^**^
			(0.099)		(0.115)
*MTD*				0.412^***^	
				(0.137)	
*MTS*					1.290^***^
					(0.275)
*ROA*	1.145^*^	0.888	0.903	0.970	0.990
	(0.693)	(0.679)	(0.679)	(0.690)	(0.679)
*Growth*	−0.202^**^	−0.206^**^	−0.213^**^	−0.238^**^	−0.153
	(0.102)	(0.104)	(0.101)	(0.104)	(0.102)
*Size*	0.489^***^	0.416^***^	0.407^***^	0.449^***^	0.393^***^
	(0.028)	(0.030)	(0.030)	(0.030)	(0.030)
*Tobin’s Q*	0.007	−0.007	−0.012	0.004	−0.015
	(0.028)	(0.028)	(0.028)	(0.028)	(0.028)
*Lev*	−1.160^***^	−1.253^***^	−1.212^***^	−1.175^***^	−1.189^***^
	(0.235)	(0.232)	(0.232)	(0.233)	(0.229)
*Property, Industry, Year*	*Control*	*Control*	*Control*	*Control*	*Control*
Obs.	1,625	1,625	1,625	1,625	1,625
*Adj R^2^*	0.354	0.380	0.390	0.382	0.378
*F*	27.862	29.515	32.532	27.928	28.319

The standard errors are in the parentheses. ^***^p < 0.01; ^**^p < 0.05; ^*^p < 0.1.

Hypotheses 1a and 1b assume that higher degree centrality and larger structural indexes in management talent flow networks can improve firms’ innovation. As shown in Column (2) of Table 3, firms’ degree centrality in the management talent flow networks (*MDC*) has significant positive effect on their innovation performance (*b* = 0.802, *p* < 0.01). Meanwhile, firms’ structural hole indexes in the management talent flow network (*MSHD*) also promotes firms’ innovation (*b* = 0.381, *p* < 0.01). That is, the regression results validate Hypotheses 1a and 1b. Hypothesis 1 of the current study is supported.

Hypotheses 2a and 2b propose that higher degree centrality and larger structural hole indexes in the technical talent flow networks are positively associated with firms’ innovation performance. As reported in Column (3) of Table 3, firms’ degree centrality in the technical talent flow networks (*TDC*) has prominent positive impact on firms’ innovation (*b* = 0.599, *p* < 0.01), and firm’ structural hole indexes in the technical talent flow networks (*TSHD*) enhances firms’ innovation as well (*b* = 0.464, *p* < 0.01). That is, the results are in favor of Hypotheses 2a and 2b. Hypothesis 2 of this study is supported.

Hypotheses 3a and 3b propose that the interactions of firms’ degree centrality and structural hole indexes in the management talent flow networks and the technical talent flow networks can improve their innovation, respectively. As revealed in Column (4) of Table 3, the interaction between firms’ degree centrality in the two networks (*MTD*) is positively associated with their innovation performance (*b* = 0.412, *p* < 0.01). And Column (5) of Table 3 shows that, the interaction between firms’ structural hole indexes in the two networks (*MTS*) also has significant positive influence on their innovation (*b* = 1.290, *p* < 0.01). Hypotheses 3a and 3b are both validated. That is, Hypothesis 3 is supported as well.

#### Balanced embeddedness in dual talent flow networks and firms’ innovation

Table 4 reports the results of one way ANOVA (the other variables are not controlled) between balanced degree centrality, balanced structural hole indexes in management talent flow networks and technical talent flow networks on firms’ innovation performance. It validates Hypotheses 4a and 4b.

**Table 4:** One way ANOVA between dual network embedding balance and firms’ innovation.

	*MTDB = 1*	*MTDB = 0*	*F*	*MTSB = 1*	*MTSB = 0*	*F*
	Column (1)	Column (2)		Column (3)	Column (4)	
*INNO*						
*Mean*	3.201	2.991	3.730^*^	3.293	2.789	30.780^***^
*Std.*	1.613	1.478		1.662	1.301	
*n*	1,371	254		1,222	403	

The standard errors are in the parentheses.

*** *p* < 0.01;

** *p* < 0.05;

* *p* < 0.1.

As shown in Column (1) and (2) of Table 4, the mean value of innovation is 3.201 for the group with balanced degree centrality in the two networks. For the group with imbalanced degree centrality in the two networks, the mean value of innovation is 2.991. The F statistic of the mean difference between the two groups is 3.730, and the correlation is significant at the 0.1 level. This suggests that, with the other variables uncontrolled, the innovation of the group with balanced degree centrality significantly excels the group with imbalanced degree centrality. Hypothesis 4a is tentatively supported.

In Column (3) and (4) of Table 4, the mean value of the innovation is 3.293 for the group with balanced structural hole indexes in the two networks. For the group with imbalanced structural hole indexes, the mean value of innovation is 2.789. The F statistic of the mean difference between the two groups is 30.780, and the correlation is significant at the 0.01 level. This indicates that, with the other variables uncontrolled, the innovation of the group with balanced structural hole indexes exceeds the group with imbalanced structural hole indexes. This tentatively supports Hypothesis 4b.

To guarantee the rigor of the results, we will introduce several control variables and conduct further multivariate analyses.

Table 5 reports the results of multivariate analyses. Hypotheses 4a and 4b are tested with the control variables included. With reference to the method outlined by Tsui et al. (1997), we add the control variables and use co-variance analyses to test Hypotheses 4a and 4b. As shown in Column (2) of Table 5, between the group with balanced degree centrality in the two networks and the group with imbalanced degree centrality, the innovation performance is significantly different (*F* = 7.460, *p* < 0.01). Furthermore, Column (3) of Table 5 shows that, between the group with balanced structural hole indexes in the two networks and the group with imbalanced structural hole indexes, the innovation outcomes also differ prominently (*F* = 13.060, *p* < 0.01). Hypotheses 4a and 4b are supported again.

**Table 5:** Covariance analysis of dual network matching effect on firms’ innovation.

Variables	Innovation performance (*INNO*)		
	Column (1)	Column (2)	Column (3)
*MTDB*		7.460^***^	
eta^2^		0.690	
*MTSB*			13.060^***^
eta^2^			0.629
*ROA*	3.170^*^	0.410	3.020^**^
*Growth*	3.330^*^	8.890^***^	2.590
*Size*	384.550^***^	609.500^***^	370.580^***^
*Tobin’s Q*	0.040	3.660^*^	0.020
*Lev*	25.450^***^	21.560^***^	27.250^***^
*Property, Industry, Year*	*Control*	*Control*	*Control*
*Obs.*	1,625	1,625	1,625
*Adj R_2_*	0.295	0.301	0.359
*F*	44.060^***^	42.940^***^	31.280^***^

The standard errors are in the parentheses.

*** *p* < 0.01;

** *p* < 0.05;

* *p* < 0.1.

## Conclusion and discussion

This paper uses online resume data to investigate the complex effects of management talent flow networks and technical talent flow networks on firms’ innovation from the social network perspective. The empirical results suggest that: (1) when firms possess central positions and abundant structural holes in both management and technical talent flow networks, their innovation improves; (2) firms’ degree centrality in the two types of networks also have mutual reinforcing effects on their innovation performance; (3) firms’ Structural hole index in the two types of networks also have mutual reinforcing effects on their innovation; (4) in terms of network embedding, firms that can balance their degree centrality and structural holes in the two types of networks, respectively, can achieve better innovation performance compared to those that can not maintain such balance. That is, advantageous embedding structures in both the management and technical talent flow networks can benefit firms’ innovation performance, and show significant interaction and matching effects simultaneously. They are mutually reinforcing and dependent.

This study mainly has two theoretical contributions: (1) It deepens the research on talent flow network and firms’ innovation performance by broadening the assumption of homogeneous talent in the existing literature on talent flow networks. In addition, as management talent flow networks and technical talent flow networks both have important impact on firms’ innovation, this paper incorporates them into one analytical framework. Then, based on the social network theory and human capital theory, it proposes the idea of conducting classification research about talent flow networks according to talent types. By constructing an interaction latitude and balance latitude of management and technical talent flow networks and examining their effects on firms’ innovation, this paper finds that, the two types of networks have complex interaction and matching effects on firms innovation. This may refresh our perceptions about the relationship between talent flow network and firms’ innovation; (2) the study examines and deepens our knowledge about the theory of total innovation management. This paper builds up the dual talent flow network based on the difference between human capital types. It also conducts in-depth research on the important effects of balanced embeddedness in management and technical talent flow networks over firms’ innovation performance. It is found that management and technical factors not only have interaction effects, but also show significant matching effect in firms’ innovation processes. This finding will promote the academic cognition about the theory of total innovation management.

This paper has two management implications: (1) By broadening the external sources of management and technical talent, increasing organizational attractiveness and enriching the talent pool, firms can occupy advantageous positions in the talent flow networks. These positions include central nodes and structural holes in the talent flow networks, which can enhance firms’ innovation performance. The empirical results of our study prove that taking advantageous structural positions in the management talent flow networks and technical talent flow networks helps to improve firms’ innovation performance. Hence, if firms intend to enhance their innovation by means of talent flow network, they need to occupy the central nodes and structural holes in both the two types of networks. If firms want to improve their degree centrality and structural hole index in the network, they should try to establish rich and heterogeneous connections with the external organizations *via* talent mobility. This means that in practice, firms must broaden the talent sources, promote their attractiveness to the talent by all means to expand their talent pool. Accordingly, they will be able to enhance their positions in the talent flow networks more precisely and efficiently; (2) while improving innovation *via* talent flow networks, firms need to maintain balance between the acquisition of management and technical talent. They should also enhance the absorption, integration and utilization of the innovative resources brought by the new hires so as to improve the innovation to the fullest extent. The empirical results of this paper indicate that, management talent flow networks and technical talent flow networks can mutually reinforce the promotion of firms’ innovation. Furthermore, firms that can balance their degree centrality and structural holes in both networks can achieve better innovation performance compared to those that can not maintain the balance. This suggests that, on the one hand, firms need to strengthen the absorption and utilization of the newly acquired management and technical resources to activate synergy. On the other hand, they should balance the acquisition of management and technical talent to maximize their innovation outcomes.

## Limitations and future research directions

This research has some limitations that should be addressed in future studies. First, upgraded anti-crawler technology in the professional networks means that this study can only access to resume data from before 2016. While most of the existing literature is based on historical data (Godart et al., 2014; Shipilov et al., 2017), and we try our best to control year effects on the results, the possibility exists that the timeliness of data might affect the robustness of the research results. Further studies should expand the data size and source so that the robustness of research conclusions may be verified. Second, based on the secondary data we obtained, it is hard to investigate the mechanism of the influencing process and examine the potential situational factors. Thus, future researches might combine secondary data and questionnaire data. A text analysis method can also be applied to identify some intermediary factors and situational factors. At last, the limited functions of data processors mean that we can only recognize the technical talent and management talent who significantly influence firms’ innovation. However, marketing talent and some other non-technical talent may also act on the innovation of firms. Therefore, future studies may seek for more advanced methods to study the complex effects of different types of talent on firm’s innovation performance.

The rapid development and wide application of information technologies such as big data, cloud computing, block-chain and artificial intelligence are reshaping the management models of various industries, the external market of enterprises and their internal resources and capabilities (Tariq et al., 2021; Centobelli et al., 2021a,b; Cerchione et al., 2022). In this context, these digital technologies have also begun to be applied to the management of talent in enterprises. Examples include using block-chain technology to ensure the authenticity of talent resumes, using artificial intelligence technology to select employees suitable for the enterprise, and using big data technology to handle the information and behavior of employee communication (Stone et al., 2015). Digital technologies and green management may also have a certain impact on the talent flow management of enterprises. For instance, management talent and technical talent can bring management and technical resources to firms. However, firms may not necessarily achieve good innovation performance until the resources have been effectively absorbed and transformed., This raises the question as to whether the new digital technology used to strengthen internal communication and cooperation will contribute to the innovation performance of enterprises. Furthermore, if firms practice green human resource management in the hiring process, will online recruiting or the so called “smart recruiting” help firms acquire more suitable talent, thereby promoting their efficiency to obtain critical innovative resources through talent flow network? Future studies should explore how digital technology and green management practice affect the relationship between talent flow networks and enterprise innovation.

## Data availability statement

The original contributions presented in the study are included in the article/supplementary material, further inquiries can be directed to the corresponding author.

## Author contributions

BS and BP: contributed to the conception and design of the research. They drafted the work and revised it critically for important intellectual content. AR and BS: contributed to the acquisition, analysis, and interpretation of data for the work. They were also responsible for the approval of the version to be published. SL: contributed to the analysis and interpretation of data for the work. All authors have made substantial contributions to the article and approved the submitted version.

## Funding

National Natural Science Foundation of China (72002075, 71832003), Philosophy and social science planning project of Guangdong Province (GD21CLJ02).

## Conflict of interest

The authors declare that the research was conducted in the absence of any commercial or financial relationships that could be construed as a potential conflict of interest.

## Publisher’s note

All claims expressed in this article are solely those of the authors and do not necessarily represent those of their affiliated organizations, or those of the publisher, the editors and the reviewers. Any product that may be evaluated in this article, or claim that may be made by its manufacturer, is not guaranteed or endorsed by the publisher.
